# Medical imaging in cancer cachexia

**DOI:** 10.1007/s00117-024-01346-5

**Published:** 2024-07-12

**Authors:** Hyun Soo Ko, Ulrike Attenberger

**Affiliations:** 1 Department of Cancer Imaging, Peter MacCallum Cancer Centre, Melbourne, VIC Australia; 2 The Sir Peter MacCallum Department of Oncology, The University of Melbourne, Melbourne, VIC Australia; 3 Department of Diagnostic and Interventional Radiology, University Hospital Bonn, Bonn, Germany

**Keywords:** Atrophy, muscle, Wasting syndrome, Radiology, Computed tomography, Imaging biomarkers, Muskelatrophie, Auszehrungssyndrom, Radiologie, Computertomographie, Bildgebungsbiomarker

## Abstract

Cancer cachexia, often referred to as “wasting syndrome,” is characterized by fatigue, weakness, and involuntary weight loss. This syndrome is concomitant with progressive skeletal muscle atrophy with or without adipose tissue loss and is frequently accompanied by systemic inflammation. Understanding the complexities of cancer cachexia is crucial for early detection and intervention, and it is also paramount for enhancing patient outcomes. Medical imaging, comprising diverse imaging modalities, plays a pivotal role in this context, facilitating the diagnosis and surveillance assessment of both the disease extent and the body composition changes that offer valuable information and insights into disease progression. This article provides a comprehensive discourse of the pathophysiological mechanisms and clinical manifestations of cancer cachexia as well as the role of medical imaging in this setting. Particular emphasis is placed on contemporary multidisciplinary and translational research efforts for the development of diagnostic and treatment tools, aiming to mitigate the devastating consequences of cancer cachexia.

## Abstract (de)

Tumorkachexie wird häufig als „Auszehrungssyndrom“ bezeichnet und ist charakterisiert durch Fatigue, Schwäche und ungewollte Gewichtsabnahme. Dieses Syndrom tritt begleitend bei progressiver Skelettmuskelatrophie mit oder ohne Fettgewebsverlust auf und geht oft mit systemischer Entzündungsreaktion einher. Das Verständnis der Komplexität der Tumorkachexie ist entscheidend für die rechtzeitige Erkennung und Behandlung und von zentraler Bedeutung für die Verbesserung der Patientenergebnisse. In diesem Zusammenhang spielt die medizinische Bildgebung mittels diverser Bildgebungsmodalitäten eine ausschlaggebende Rolle. Sie erleichtert die Diagnosestellung und die Kontrolluntersuchungen sowohl des Krankheitsausmaßes als auch der Veränderung der Körperzusammensetzung, die wertvolle Informationen und Einsichten in den Krankheitsprogress ermöglichen. In der vorliegenden Arbeit wird eine umfassende Erörterung der pathophysiologischen Mechanismen und klinischen Manifestationen der Tumorkachexie sowie der Bedeutung der medizinischen Bildgebung in diesem Rahmen gegeben. Besonderer Wert wird auf die aktuellen multidisziplinären und translationalen wissenschaftlichen Bestrebungen für die Entwicklung diagnostischer und therapeutischer Instrumente gelegt, die darauf abzielen, die verheerenden Folgen der Tumorkachexie abzumildern.

## Introduction

Cachexia is a multifactorial syndrome characterized by unintentional weight loss, weakness, and fatigue due to progressive skeletal muscle wasting, with or without adipose tissue loss [1]. It differs from starvation, as it is not reversible upon increased calorie intake [2]. Up to 80% of advanced cancer patients are affected, which accounts for up to one third of cancer-related mortality [3]. It causes a commonly overlooked tumor-induced systemic inflammation and cross-organ dysfunction leading to reduced tolerance to cancer treatments, decreased quality of life, and increased mortality [1].

## Clinical manifestations and pathophysiology of cancer cachexia

Clinical manifestations of cancer cachexia comprise a combination of symptoms with the hallmark presentation of progressive weight and skeletal muscle loss with or without adipose tissue depletion [1]. Cancer cachexia is a continuum of symptoms that can vary according to type, location, and size of the cancer. It differs from starvation, as increased caloric intake cannot restore this weight loss. Furthermore, the fact that it can be dependent on the treatment efficacy distinguishes it from age-related sarcopenia and frailty [2, 4].

Cachexia can also be linked to non-cancerous etiologies, such as diabetes, autoimmune, chronic cardiac, pulmonary, kidney, and liver diseases [5, 6]. Despite their similar clinical manifestations, cancer and non-cancer-related cachexia differ in terms of underlying disease pathophysiology, onset, disease progression, and prognosis [1, 2, 7].

The prevalence of cancer cachexia varies according to the malignancy type. For example, the prevalence rate in gastroesophageal and pancreatic cancer is around 70% and in lung and colorectal malignancies it ranges from 40% to 50%, whereas in prostate and breast cancer the rate is estimated to be approximately 20% [8]. This also correlates with patient outcomes, such as overall survival [9].

The three clinical stages of cancer cachexia are precachexia, cachexia, and refractory/advanced cachexia; however, not all three stages necessarily occur during a patient’s journey [1, 4]:

- *Precachexia* is an early stage of cachexia that is characterized by systemic inflammation and unintentional loss of ≤ 5% of weight [4]. At this early stage of disease, cachexia is reversible; however, as the symptoms are not easily recognized and detectable, the disease usually progresses further [1, 4].
- The *cachexia* stage is defined by systemic inflammation and unintentional weight loss of > 5% in the preceding 6 months in patients with a normal or high body mass index (BMI), or of > 2% in sarcopenic (skeletal muscle index: males < 7.26 kg/m^2^, females < 5.45 kg/m^2^) or underweight patients (BMI < 20 kg/m^2^), who have not entered the refractory/advanced stage of cachexia [1, 4].
- By the time the patients reach the *refractory/advanced cachexia* stage, they no longer respond to treatment. This stage is correlated with very advanced cancer, with an expected survival of less than 3 months [1, 4].

Cancer cachexia is thought to be mediated by complex crosstalk between the tumor and peripheral tissues, which in turn leads to metabolic and systemic changes. These effects can be caused by the tumor directly or by paraneoplastic-mediated effects as well as through systemic inflammation [1].

Systemic inflammation includes pro-inflammatory cytokines such as tumor necrosis factor-alpha (TNF-α), interleukin‑6 (IL-6), and interleukin‑1 beta (IL-1β; [10]). These cytokines inhibit muscle protein synthesis and promote muscle protein and adipocytes breakdown, leading to the release of free fatty acid, glycerol, and triglycerides. Other muscle catabolic drivers are myostatin and activins, which are members of the transforming growth factor-beta (TGF-β) superfamily [11]. Elevated levels of myostatin and activins contribute to muscle wasting by inhibiting muscle protein synthesis and promoting muscle protein breakdown [12]. Furthermore, impaired mitochondrial cellular energy production leads to muscle weakness and fatigue in cancer cachexia. This mitochondrial dysfunction may stem from increased reactive oxygen species (ROS) production, impaired mitochondrial biogenesis, and altered mitochondrial dynamics [9, 13]. Another well-known pathway of intracellular protein degradation is the ubiquitin-proteasome system (UPS), and increased cellular autophagy is also implicated in cancer cachexia [12].

In addition, genetic and other factors that dictate an array of physiological processes including the circadian rhythm, food intake stimuli, and endocrine functions have been proven to influence tissue crosstalk as demonstrated in multiple animal models of cancer cachexia [14, 15]. Cachexia also induces changes that include insulin resistance, impaired glucose metabolism, and dyslipidemia, which further contribute to fatigue and weakness.

Fatigue can also be a result of anorexia or reduced food intake because of a decreased appetite, chemosensory disturbances (e.g., food smell and taste), and upper or lower gastrointestinal tract dysmotility (e.g., nausea, early satiety, constipation; [4, 16]). Fatigue may be disproportionate to the level of physical activity and is often not alleviated by rest.

Together these mediators of cancer cachexia cause a multitude of symptoms that impact the quality of life of affected individuals, leading to physical, emotional, and psychosocial distress [4, 16]. Patients may experience anxiety, social isolation, decreased self-esteem, and depression, further aggravating the burden of the disease.

Despite continuous increase in research and clinical measures to fight these devastating consequences of cancer cachexia, this entity often remains unnoticed and therefore untreated.

## Role of medical imaging in cancer cachexia

Clinical and imaging biomarkers offer options for early detection and continued monitoring of cancer cachexia. Therefore, they are promising approaches to reduce the prevalence of cancer cachexia, and to increase treatment tolerance and response to cancer therapy. In addition, novel approaches to enable early detection will ultimately result in increased treatment options, and thus improve patient outcomes. So far, cachexia is mostly defined according to BMI, which does not offer a full picture of wasting.

**Fig. 1:**
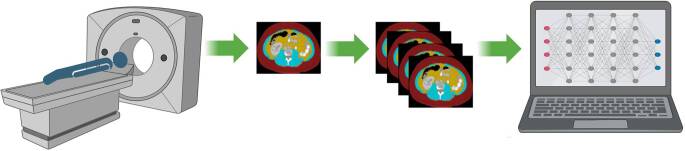
Pipeline of noninvasive screening tool for cancer cachexia, incorporating opportunistic artificial intelligence-assisted imaging analysis of body composition. (Adapted from [17])

Radiological and nuclear medicine imaging is vital for the diagnosis and surveillance of cancer patients, providing essential information on disease extent and other relevant findings, such as surgical complications and treatment toxicity. An emerging avenue to potentially establish itself as a cornerstone of standard-of-care involves leveraging noninvasive imaging-derived body composition biomarkers obtained from cross-sectional examinations (Fig. 1; [17]).

**Fig. 2:**
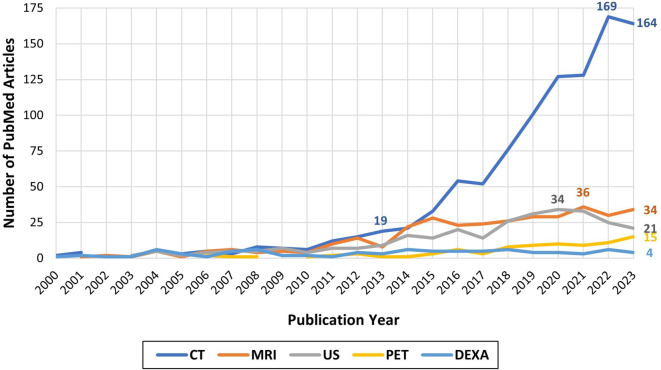
Number of PubMed articles on body composition, cancer, and the most common medical imaging modalities from 2000 to 2023. *CT* computed tomography, *MRI* magnetic resonance imaging, *US* ultrasound, *PET* positron emission tomography, *DEXA* dual-energy X‑ray absorptiometry

Among the medical imaging modalities available, computed tomography (CT) emerges as the clear predominant imaging examination employed since 2000 (Fig. 2). Over the past decade there was an over eightfold increase in publications indexed in PubMed pertaining to CT, body composition, and cancer (19 in 2013 and 164 in 2023). This trend is attributed to recent advances in machine and deep learning artificial intelligence (AI) big data analysis derived from routinely performed examinations, also referred to as “opportunistic” scanning [18]. Indexed research studies of body composition utilizing other modalities demonstrate a comparatively lower frequency, with investigations utilizing magnetic resonance imaging (MRI), ultrasound (US), and positron emission tomography (PET) showing a marginally elevated level of research attention over the past decade, yielding as many as 36 published articles annually. By contrast, a lower publication rate is found for studies employing dual-energy X‑ray absorptiometry (DEXA), averaging fewer than ten articles per year.

### Computed tomography

**Fig. 3:**
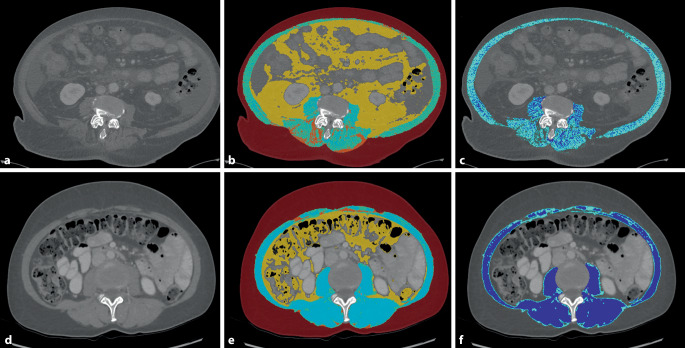
Body composition analysis at the L3/L4 lumbar level and tissue segmentation in two patients with pancreatic ductal adenocarcinoma. **a–c** Imaging of an 81-year-old male patient with cachexia showing low muscle attenuation and low muscle area as well as low subcutaneous and high visceral fat area. **d–f** Imaging of a 49-year-old female patient without cachexia showing high muscle attenuation and area as well as high subcutaneous and low intramuscular fat area. **a,** **d** CT source image. **b,** **e** CT image with body composition overlay (*light blue*: skeletal muscle, *yellow*: visceral fat, *maroon*: subcutaneous fat, *orange*: intermuscular fat). **c,** **f** CT image with skeletal muscle body compartment overlay (*dark blue*: lean muscle, *light blue*: intramuscular fat, *olive*: intermuscular fat)

Computed tomography is widely available and the most employed modality to quantify muscle and adipose tissue distribution, enabling an accurate detection of loss of muscle and adipose tissue. In addition, increased open-source software, AI codes, and imaging data repository utilization are providing new opportunities for detecting predictive imaging biomarkers in cancer cachexia [19]. In general, CT body composition analysis is usually performed at the L3 vertebral level, which is included in most chest and abdomen CT scans (Fig. 3). This single-slice two-dimensional (2D) approach has shown a good correlation to multislice volume analysis [19]. Other cross-sectional vertebral levels, such as C3, C5, and L1, have also been of interest in providing representative body composition parameters, which could be useful when the field of view scanned does not encompass the L3 vertebral level [20, 21].

Depending on the cancer type, various body composition features have been suggested as imaging biomarkers: For example, Xu et al. showed subcutaneous adipose and skeletal muscle attenuation to add predictive value in lung cancer-related deaths within a large CT lung screening cohort (> 20,000 patients; [22]), and Lee et al. showed skeletal muscle attenuation, subcutaneous fat area, and aortic calcium to be predictive for colorectal cancer survival [23].

### Magnetic resonance imaging

Magnetic resonance imaging can be used to evaluate cancer cachexia body composition changes, providing high-resolution images and therefore more detailed insights regarding muscle and adipose soft tissue qualities. As with CT, the examination commonly utilizes L3 vertebral body single-slice body composition analysis [24]. However, single-slice data extractions from alternative anatomical regions, such as the mid-femur or inframammary subcutaneous abdominal fat, have been suggested as potential opportunistic predictive biomarkers for evaluating skeletal and adipose tissue loss [25]. This suggestion is exemplified in an MRI breast study, which demonstrated an association between breast cancer and the thickness of the upper abdominal adipose layer, serving as a surrogate marker for body adiposity among breast cancer patients [26].

### Dual-energy X-ray absorptiometry

Dual-energy X‑ray absorptiometry scans are valuable for assessing body composition by measuring adipose tissue, lean muscle tissue mass, and in particular bone mineral density [9]. However, measurements are highly scanner dependent, resulting in poor uptake of research employing DEXA, as shown in Fig. 2. Furthermore, recent advances in comparing CT-based bone density measurements with overall morbidity (e.g., risk of fracture) and overall mortality might lead to DEXA being replaced by CT [23].

### Ultrasound

Ultrasound can serve as a tool for assessing muscle thickness and quality, which is useful in settings with limited resources or logistical constraints, such as intensive care units or practices with restricted access to other cross-sectional imaging modalities [27]. An example of a targeted US application for assessing muscle quantity and function has been described by Chu et al., demonstrating an increase in post-exercise quadriceps femoris and biceps brachii/brachialis muscle thickness and echogenicity among metastatic breast cancer patients [28]. Nonetheless, disadvantages include notable inter-user variability and the non-opportunistic nature of US, impeding large-scale data accrual and subsequent poor generalizability of body composition measurements derived from US examinations.

### Positron emission tomography

Positron emission tomography assesses metabolic activity and thereby detects tumors and metastases that contribute to the development of cancer cachexia. Furthermore, PET is commonly utilized to detect infection; however, to date, there are no current studies evaluating the role of PET in the detection of infection in cancer patients affected by cachexia. A possible added value of PET diagnostics could include opportunistic evaluation of potential cancer-associated metabolic changes, as shown in a study of 132 cancer patients with varying tumor types, in which a higher volume of 18F-FDG PET dark brown adipose tissue was associated with higher tumor recurrence and mortality [29].

Moreover, with increased theranostic radiotracer development, novel metabolic target discoveries could provide new treatment pathways to fight cancer cachexia.

Usually, PET is performed in conjunction with CT/MRI to increase spatial accuracy, and these adjunct modalities can certainly be utilized for body composition analysis as mentioned earlier [30].

## Future directions in cancer cachexia

The aim of forthcoming strategies aimed at combating cancer cachexia necessitates a multifaceted approach, encompassing diverse methodologies ranging from laboratory investigations to comprehensive clinical research aimed at elucidating the pathophysiological mechanisms and identifying key clinical determinants to develop effective diagnostic and therapeutic tools [17].

Muscle strength weakness and diminished muscle mass are often based on all the aforementioned factors, with muscle strength being measured through functional parameters, such as upper-limb hand grip dynamometry, and with skeletal muscle mass being increasingly assessed with cross-sectional imaging [1].

Current medical imaging techniques and interpretations of cancer cachexia are facing significant limitations, and to date they have not been integrated into standard of care, despite considerable research efforts in the field of imaging-based body composition analysis [17]. This can be attributed to the lack of awareness of cancer cachexia among clinicians, such as radiologists and referring doctors as well as patients and their caregivers. Additionally, there remains a notable absence of international consensus guidelines or high-level evidence-based research that hinders the establishment and dissemination of protocols. Nevertheless, translational research in medical imaging holds considerable promise for the identification of predictive biomarkers that, when integrated with conventional clinical and laboratory parameters, could establish new standards of care, thereby enhancing the quality of life and overall survival rates among oncological patients [17].

The availability of opportunistic imaging-derived body composition analysis presents a noninvasive avenue for facilitating the diagnosis and monitoring of cancer cachexia. Future predictive imaging biomarkers will likely vary depending on the type of malignancy. However, CT-derived skeletal muscle and subcutaneous and visceral fat densities, as well as their respective areas/volumes, are high contenders to be integrated in future prognostic modeling [21–23]. Furthermore, adding and combining clinical and imaging parameters to the modeling will add the multidisciplinary decision-making value [19, 22, 23]. This could be particularly impactful in smaller practice settings that have limited access to specialized multidisciplinary care. Another research field is called “radiomics,” which encompasses the computational extraction of pixel/voxel relationships from medical imaging. This has the potential to offer additional pathways for the development of translational imaging biomarkers. Numerous software platforms and methodological approaches in the field of body composition analysis and radiomics are currently undergoing development, with a growing incorporation of AI technologies. The aim is to improve or even replace current cancer cachexia metrics and definitions, by developing novel predictive models that could include imaging-derived biomarkers [9, 17]. Imaging assessment of cancer cachexia will depend on the modality usage and frequency, which are specific to the respective indication and often dependent on the cancer type and stage. Therefore, future prognostic imaging biomarkers will likely be derived from CT, followed by PET-CT and MRI.

With the increasing utilization of AI and the collection and analysis of big data, larger and more diverse cohort sizes are becoming more feasible. This has the potential to improve early cancer cachexia detection, more accurate diagnosis, and monitoring, therefore enabling successful integration of supportive care interventions into routine oncology practice.

Other necessary initiatives involve the establishment of specialized multidisciplinary cancer cachexia clinics, comprising a collaborative team with radiologists and nuclear medicine physicians being well embedded within the group. This approach would allow for the development and execution of randomized and multicenter trials, early-detection strategies, and targeted and improved treatment interventions [4, 16, 17]. These central hubs could integrate research, education, and clinical care, enabling the development of new gold-standard measures, protocols, and guidelines regarding the detection and treatment of cancer cachexia. Additionally, they could foster increased awareness of cancer cachexia by disseminating information within, and providing training to, the broader community [17].

## Practical conclusion

- Clinical manifestations of cancer cachexia are multifaceted, reflecting the complex interplay of metabolic, inflammatory, and neuroendocrine factors involved in the pathogenesis of the condition.
- Cancer cachexia differs from starvation, as it is not reversible upon increased calorie intake.
- Cancer cachexia affects up to 80% of advanced cancer patients.
- Cancer cachexia has three linear stages: precachexia, cachexia, and refractory/advanced cachexia.
- Understanding the complex molecular mechanisms and interorgan crosstalk is crucial for the development of targeted therapies aimed at preventing or reversing cancer cachexia.
- Effective management of cancer cachexia requires a multidisciplinary strategy focused on early detection and intervention, aiming to enhance patient outcomes.
- Medical imaging and artificial intelligence play a pivotal role, with future validated body composition parameters as promising standard-of-care imaging biomarkers of cancer cachexia.

## Declarations

For this article no studies with human participants or animals were performed by any of the authors. All studies mentioned were in accordance with the ethical standards indicated in each case.

The supplement containing this article is not sponsored by industry.
